# Prediction of Three-Directional Ground Reaction Forces during Walking Using a Shoe Sole Sensor System and Machine Learning

**DOI:** 10.3390/s23218985

**Published:** 2023-11-05

**Authors:** Takeshi Yamaguchi, Yuya Takahashi, Yoshihiro Sasaki

**Affiliations:** 1 Department of Finemechanics, Graduate School of Engineering, Tohoku University, Sendai 980-8579, Japan; yuya.takahashi.p1@dc.tohoku.ac.jp; 2 Graduate School of Biomedical Engineering, Tohoku University, Sendai 980-8579, Japan; 3 Research Institute for Electromagnetic Materials, Tomiya 981-3341, Japan; sasaki@denjiken.ne.jp

**Keywords:** ground reaction force, shoe sole sensor system, machine learning, gait, walking, turning

## Abstract

We developed a shoe sole sensor system with four high-capacity, compact triaxial force sensors using a nitrogen added chromium strain-sensitive thin film mounted on the sole of a shoe. Walking experiments were performed, including straight walking and turning (side-step and cross-step turning), in six healthy young male participants and two healthy young female participants wearing the sole sensor system. A regression model to predict three-directional ground reaction forces (GRFs) from force sensor outputs was created using multiple linear regression and Gaussian process regression (GPR). The predicted GRF values were compared with the GRF values measured with a force plate. In the model trained on data from the straight walking and turning trials, the percent root-mean-square error (%RMSE) for predicting the GRFs in the anteroposterior and vertical directions was less than 15%, except for the GRF in the mediolateral direction. The model trained separately for straight walking, side-step turning, and cross-step turning showed a %RMSE of less than 15% in all directions in the GPR model, which is considered accurate for practical use.

## 1. Introduction

With the advent of aging in society, the number of falling accidents among elderly people and patients with gait disorders is increasing. Thus, fall prediction and rehabilitation based on balance assessment are becoming increasingly important. The measurement of ground reaction forces (GRFs) during gait is used in a wide range of medical and healthcare applications, including the assessment of fall risk [1], diagnosis of pathological gait [2], and biofeedback for rehabilitation [3]. Three-directional GRFs are usually measured using force plates because of their high accuracy. However, force plates cannot be easily introduced in small clinical facilities owing to their high cost, and space and movement limitations. Furthermore, it is difficult to measure GRFs with force plates during natural gait in a living environment.

Many attempts have been made to measure GRFs without using force plates. Studies have been conducted to estimate GRFs using kinematic data obtained from marker-less motion capture [4,5]. Liu et al. [6] found that if the cross-correlation coefficient is greater than 0.9 and the percent root-mean-square error (%RMSE) is less than 15%, the accuracy of GRF estimation is excellent. However, limited reports using marker-less motion capture met these criteria for GRF estimation in three-axis directions. Another problem is that the use of a camera also imposes location restrictions. GRF estimation using wearable sensors equipped with inertial measurement units (IMUs) is widely adopted because of its ease of use and low cost, and it is less subject to location and motion restrictions [7,8,9]. One potential problem with an IMU system is that its performance can be reduced by placement error [10]. Shoe sole sensor systems incorporating devices that measure forces in shoes, such as pressure sensors embedded in insoles and triaxial force sensors attached to the outer sole of shoes, have been developed. Moreover, various types of low-cost in-shoe mobile measurement systems using a small number of force sensors have been developed in previous studies [11,12,13,14,15]. Furthermore, some groups have proposed the estimation of GRFs during walking from these local measurements, using machine learning techniques, such as linear [16,17,18] and nonlinear [19,20,21] regressions. The insole-type sensor uses only vertical forces to estimate three-directional GRFs, and a three-axis force sensor would enable estimation with the addition of horizontal (anteroposterior and mediolateral) force data, which could increase accuracy. Therefore, shoe sole sensor systems with three-axis force sensors attached to the outer sole of the shoe have been developed [22,23]. Although these systems are superior to previous systems as they can directly measure GRFs and have a small error rate, the possibility that they may affect natural gait cannot be denied. Moriyasu et al. [24] and Yamaguchi [25] developed sole sensor systems with a number of small triaxial force sensors mounted on the outer sole of a shoe to measure GRF distribution during running and walking, respectively, but the systems had problems, such as an inability to measure the vertical GRF component at the heel, where a high load is applied owing to the limited rated capacity of the sensors.

In the present study, we developed a lightweight sole sensor system with a high-capacity, compact triaxial force sensor using a nitrogen added chromium (Cr–N) strain-sensitive thin film [26,27] mounted at four points (heel, fifth metatarsal, first metatarsal, and toe) on the sole of a shoe, and estimated three-directional GRFs using machine learning. We aimed to investigate whether it is possible to estimate three-directional GRFs using machine learning, such as multiple linear regression (MLR) analysis and Gaussian process regression (GPR), with a small number of data measured using the sole sensor system. In addition, since it is difficult to obtain a large amount of data when considering the application to clinics, we used a GPR model [28,29,30,31] and cross-validation to enable estimation without overfitting even with a small amount of data.

## 2. Methods

### 2.1. Sole Sensor System Using a Cr–N Strain-Sensitive Thin Film

**Figure 1:**
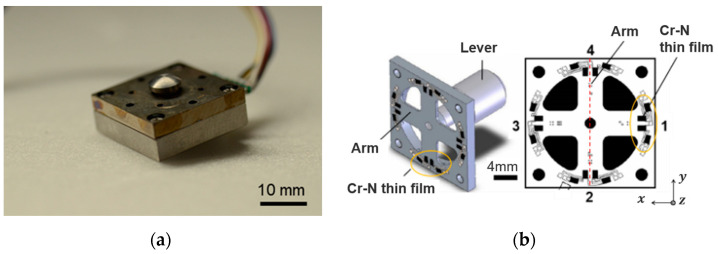
(**a**) A triaxial force sensor using a Cr–N thin film, and (**b**) schematic diagram of the structure of the sensor and layout of the Cr–N thin film.

The triaxial force sensor using a Cr–N thin film (Research Institute for Electromagnetic Materials, Tomiya, Japan; dimensions: 20 mm × 20 mm × 7.5 mm; mass: 18 g) used in this study is shown in Figure 1. The sensor consists of a 20 mm square stainless-steel housing and a force-sensing contactor (lever). The rate capacities of the sensor in the *x*, *y*, and *z* directions were ±500 N, ±500 N, and 1000 N, respectively. As shown in Figure 1b, the force sensor was fabricated by forming an insulating film on the stainless-steel strain structure and directly forming a Cr–N thin film on it through sputtering [26]. When a force acts on the tip of the contactor, voltage output signals are obtained from four sets of Cr–N strain gauge thin-film elements on the outer edge of each of the four arms, and the magnitude and direction of the applied force can be estimated by analyzing these signals [26,27].

**Figure 2:**
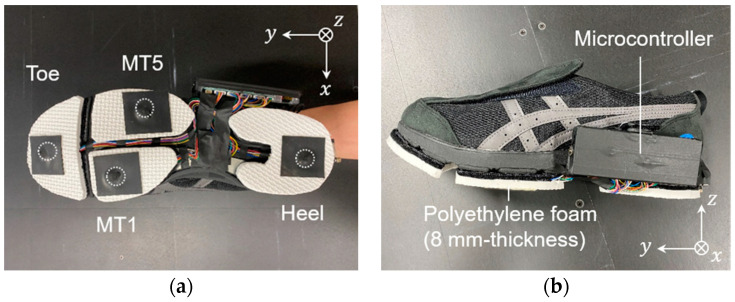
Sole sensor system. MT1 and MT5 represent the first and fifth metatarsal heads, respectively. (**a**) Location of the four triaxial force sensors, and (**b**) side view of the sole sensor system.

Figure 2 shows the appearance of the shoe sole sensor system developed in this study. An 8 mm thick polyethylene foam outsole was attached to the sole of a walking shoe (LifeWalker Men’s FLC101, size: 27.0 cm; LifeWalker Women’s FLC307, size: 25.0 cm; ASICS, Kobe, Japan). A Cr–N thin-film strain sensor was attached to the partially cut-out portion of the sole. A total of eight sensors, four for each shoe, were mounted simultaneously to measure three-directional forces at each location. The sensors were covered with 1 mm thick nitrile rubber to prevent abrasion between the contactor of the sensors and the ground surface. The force sensors were wired to a microcontroller (Teensy 3.6, SparkFun Electronics ^®^, Niwot, CO, USA), and a case containing the board and battery was attached to the side of the shoe. The mass of the entire shoe sole sensor system, including each sensor, battery, and board, was less than 380 g. Force data were recorded to an SD card for each measurement. The sampling frequency for data measurement was 870 Hz. As shown in Figure 2a, the *x*, *y*, and *z* directions of the force measured by the small triaxial force sensor were the foot width, foot length, and vertical direction of the shoe. The three-directional forces measured by the triaxial sensor were denoted as ***f****_xi_*, ***f**_yi_*, and ***f****_zi_*, respectively, where *i* denotes the position of the sensor, *i* = 1 is the heel, *i* = 2 is the first metatarsal, *i* = 3 is the fifth metatarsal, and *i* = 4 is the toe.

### 2.2. Participants

The study included six healthy young males and two healthy young females. The mean ± SD age, height, and body mass of the participants were 22.0 ± 1.7 years, 1.69 ± 0.048 m, and 58.0 ± 5.4 kg, respectively. The experimental protocol of this study was approved in advance by the Ethics Committee for Human Subjects Research, Graduate School of Engineering, Tohoku University (20A-5), and informed consent was obtained in writing from each participant after providing them with an explanation of the experimental methods and precautions in advance.

### 2.3. Experimental Procedure

**Figure 3:**
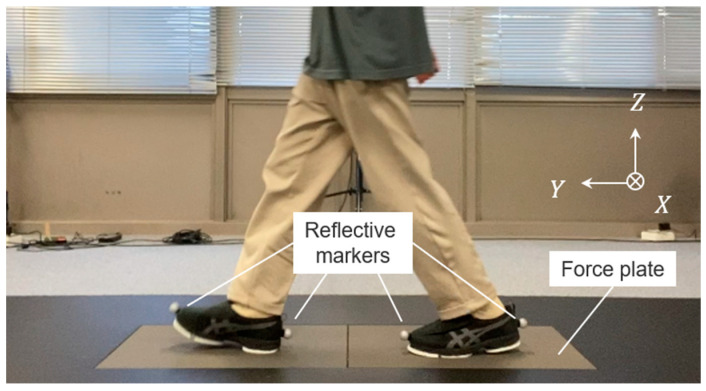
Experimental setup for gait trials, and the *X*, *Y*, and *Z* coordinates in the walking experiment system.

Participants were instructed to walk on a 5 m long walkway with two force plates (FP4060-08, Bertec, Columbus, OH, USA; each was 0.6 m × 0.4 m in size) embedded in the center (Figure 3). Infrared reflective markers were attached to the toes and heels of the sole sensor system, and the position coordinates of the infrared reflective markers were measured using a three-dimensional motion analysis system (Optitrack, Acuity Inc., Reston, VA, USA). The sampling frequency for the position data of each marker in the three-dimensional motion analysis system was 200 Hz, and the sampling frequency of the GRF data (***F****_X_*, ***F****_Y_*, and ***F****_Z_*) on the force plate was 1000 Hz. The *X*, *Y*, and *Z* coordinates in the walking experiment system were defined as shown in Figure 3, and the three-directional reaction forces measured by the force plate were denoted as ***F****_X_*, ***F****_Y_*, and ***F****_Z_*, respectively.

**Figure 4:**
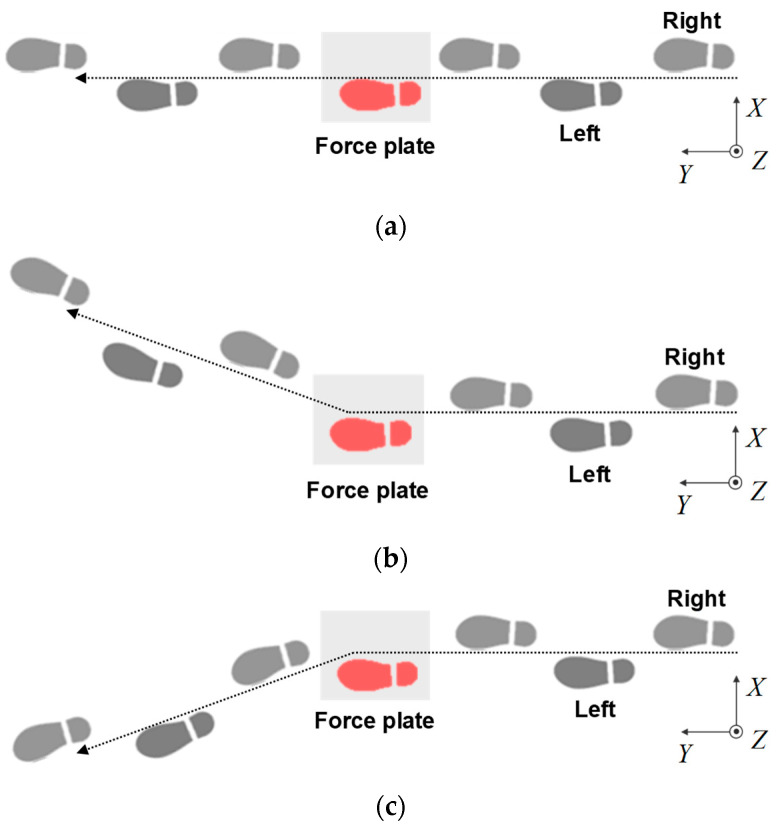
Schematic of footprints for each type of gait trial. (**a**) Straight walking; (**b**) Side-step turning; (**c**) Cross-step turning.

The participants were instructed to walk on a walkway from a stationary standing position with a self-selected stride and walking speed, and to step on the first force plate with the left foot. As shown in Figure 4, the participants were instructed to walk in a straight line and then turn on the first force plate. The participants performed two types of turns, a side-step turn (Figure 4b) and a cross-step turn (Figure 4c), as well as straight walking (Figure 4a), for a total of three types of walking styles. The turning angle was approximately 20 degrees for each turning trial. Participants performed multiple practice trials for each gait movement and performed 10 gait experiments for each gait movement.

### 2.4. Data Analysis

#### 2.4.1. Data Preprocessing

**Figure 5:**
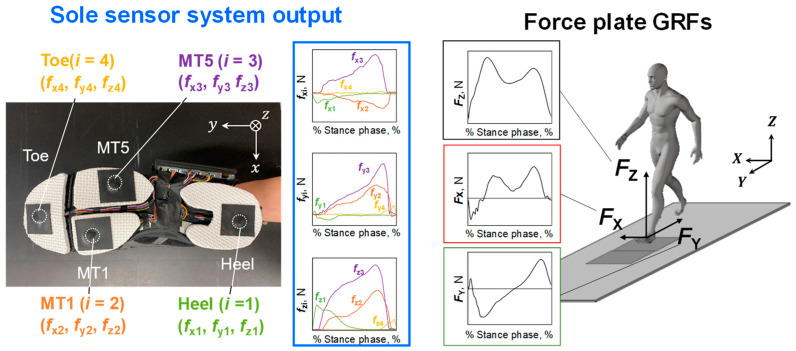
Forces obtained by the shoe sole sensor system and ground reaction forces (GRFs) obtained by a force plate. Twelve force sensor outputs (***f****_xi_*, ***f****_yi_*, ***f****_zi_* [*i* = 1–4]) were used to estimate a model to predict the GRF in each direction.

The time series data of ***f****_xi_*, ***f****_yi_*, and ***f****_zi_* (*i* = 1–4), and ***F****_X_*, ***F****_Y_*, and ***F****_Z_* during the stance phase on the first force plate in each gait trial were used for the analysis (Figure 5). Matlab ver. 9.11 (Mathworks, Natick, MA, USA) was used for subsequent analyses. The time series data were smoothed by applying a fourth-order Butterworth low-pass filter with a cutoff frequency of 50 Hz. For the total value of ***f****_zi_* (*i* = 1–4), i.e., $\overset{i=4}{\underset{i=1}{\sum }}f_{zi}$, and the time series data of ***F****_Z_*, the threshold value was set at 15 N for the sole sensor system and 50 N for the force plate system [32,33] to determine the stance phase. The angle φ of the *y*-axis of the sensor to the *Y*-axis of the force plate coordinate axis was obtained from the reflective markers attached to the toes and heels, and the coordinate transformation for the GRF components of the force plate was performed as follows:

(1) $$\left[\begin{array}{c} F_{x}\\ F_{y}\\ F_{z} \end{array}\right]=\left[\begin{array}{ccc} \cos\varphi & \sin\varphi & 0\\ -\sin\varphi & \cos\varphi & 0\\ 0 & 0 & 1 \end{array}\right]\left[\begin{array}{c} F_{X}\\ F_{Y}\\ F_{Z} \end{array}\right]$$

where ***F****_x_*, ***F****_y_*, and ***F****_z_* are the GRF components obtained from the force plate in the coordinate system (*x*-*y*-*z*) for the shoe sole sensor system. Thereafter, the time series data of both the shoe sole sensor system (***f****_xi_*, ***f****_yi_*, and ***f****_zi_*) and the force plate (***F****_x_*, ***F****_y_*, and ***F****_z_*) were reconstructed into 101 data sets (one for each 1%) by resampling, with the heel contact at 0% and the toe-off at 100%.

#### 2.4.2. Machine Learning Models

The test data ***T*** and training data ***D*** are expressed by the following equations:

(2) $$T=\left[F_{\mathrm{test}}\;|\;f_{\mathrm{test}}\right]$$

(3) $$F_{\mathrm{test}}=\left[F_{x\mathrm{test}}\;F_{y\mathrm{test}}\;F_{z\mathrm{test}}\right]$$

(4) $$f_{\mathrm{test}}=\left[f_{x1\mathrm{test}}\;f_{x2\mathrm{test}}\;f_{x3\mathrm{test}}\;f_{x4\mathrm{test}}\;f_{y1\mathrm{test}}\;f_{y2\mathrm{test}}\;f_{y3\mathrm{test}}\;f_{y4\mathrm{test}}\;f_{z1\mathrm{test}}\;f_{z2\mathrm{test}}\;f_{z3\mathrm{test}}\;f_{z4\mathrm{test}}\right]$$

(5) $$D=\left[F\;|\;f\right]$$

(6) $$F=\left[F_{x}\;F_{y}\;F_{z}\right]$$

(7) $$f=\left[f_{x1}\;f_{x2}\;f_{x3}\;f_{x4}\;f_{y1}\;f_{y2}\;f_{y3}\;f_{y4}\;f_{z1}\;f_{z2}\;f_{z3}\;f_{z4}\right]$$

(8) $$[F_{x\mathrm{test}}\;F_{y\mathrm{test}}\;F_{z\mathrm{test}}]=\left[\begin{array}{ccc} F_{x\mathrm{test}}^{1} & F_{y\mathrm{test}}^{1} & F_{z\mathrm{test}}^{1}\\ \vdots & \vdots & \vdots \\ F_{x\mathrm{test}}^{j} & F_{y\mathrm{test}}^{j} & F_{z\mathrm{test}}^{j}\\ \vdots & \vdots & \vdots \\ F_{x\mathrm{test}}^{n_{\mathrm{test}}} & F_{y\mathrm{test}}^{n_{\mathrm{test}}} & F_{z\mathrm{test}}^{n\mathrm{test}} \end{array}\right]$$

(9) $$F_{x}=\left[\begin{array}{ccc} F_{x}^{1} & F_{y}^{1} & F_{z}^{1}\\ \vdots & \vdots & \vdots \\ F_{x}^{j} & F_{y}^{j} & F_{z}^{j}\\ \vdots & \vdots & \vdots \\ F_{y}^{n} & F_{z}^{n} & F_{x}^{n} \end{array}\right]$$

(10) $$[f_{xi\mathrm{test}}\;f_{yi\mathrm{test}}\;f_{zi\mathrm{test}}]=\left[\begin{array}{ccc} f_{xi\mathrm{test}}^{1} & f_{yi\mathrm{test}}^{1} & f_{zi\mathrm{test}}^{1}\\ \vdots & \vdots & \vdots \\ f_{xi\mathrm{test}}^{j} & f_{yi\mathrm{test}}^{j} & f_{zi\mathrm{test}}^{j}\\ \vdots & \vdots & \vdots \\ f_{xi\mathrm{test}}^{n_{\mathrm{test}}} & f_{yi\mathrm{test}}^{n_{\mathrm{test}}} & f_{zi\mathrm{test}}^{n_{\mathrm{test}}} \end{array}\right]$$

(11) $$\left[f_{xi}\;f_{yi}\;f_{xi}\right]=\left[\begin{array}{ccc} f_{xi}^{1} & f_{yi}^{1} & f_{zi}^{1}\\ \vdots & \vdots & \vdots \\ f_{xi}^{j} & f_{yi}^{j} & f_{zi}^{j}\\ \vdots & \vdots & \vdots \\ f_{xi}^{n} & f_{yi}^{n} & f_{zi}^{n} \end{array}\right]$$

where ***F****_x_*_test_, ***F**_y_*_test_, and ***F****_z_*_test_ are the *x*-, *y*-, and *z*-directional components of ***F***_test_, respectively; ***f****_xi_*_test_, ***f****_yi_*_test_, and ***f****_zi_*_test_ are the *x*-, *y*-, and *z*-directional components of ***f***_test_ at each sensor position (*i* = 1–4), respectively; ***f****_xi_*, ***f****_yi_*, and ***f***_zi_ are the *x*-, *y*-, and *z*-directional components of ***f*** at each sensor position (*i* = 1–4), respectively; and *n* and *n*_test_ are the numbers of samples of the training data and test data, respectively.

Multiple linear regression (MLR) analysis was conducted using the GRFs obtained from the force plate as dependent variables and the triaxial forces obtained from the shoe sole sensor system as independent variables. The regression model is expressed by the following equation:

(12) $$\left[\begin{array}{c} {\hat{F}}_{x}\\ {\hat{F}}_{y}\\ {\hat{F}}_{z} \end{array}\right]=f_{\mathrm{test}}\left[\begin{array}{c} k_{x}\\ k_{y}\\ k_{z} \end{array}\right]+\left[\begin{array}{c} b_{x}\\ b_{y}\\ b_{z} \end{array}\right]$$

where ${\hat{F}}_{x}$, ${\hat{F}}_{y}$, and ${\hat{F}}_{z}$ are the vectors of estimated GRFs (*n*__test_ × 1) for a single step used in the test data; ***k****_x_*, ***k****_y_*, and ***k****_z_* are the vectors of partial regression coefficients (*k* × 1); ***b****_x_*, ***b****_y_*, and ***b***_z_ are vectors consisting of constant terms (*n*_test_ × 1); and *k* is the number of independent variables. Partial regression coefficients were obtained using the least-squares method, and the independent variables used in the model were determined using the stepwise method.

Predictions were also made using Gaussian process regression (GPR), a nonlinear regression widely used in time series analysis [30]. GPR provides the probability distribution of the objective variable and outputs the prediction uncertainty as standard deviation [28,29,30,31]. For example, in the *x* direction, the probability distribution of the predicted force plate GRF $p\left({\hat{F}}_{x}\right)$ is expressed in the following equation:

(13) $$p\left({\hat{F}}_{x}\right)=N\left(F_{x\mathrm{test}}{}^{*}+H\beta ,\sigma^{2}I\right)$$

where $F_{x\mathrm{test}}{}^{*}$ is the latent variable in GPR, ***H*** is a basis function vector consisting of 1, ***β*** is the vector of the coefficients of ***H***, *σ* is the noise standard deviation, and ***I*** is the unit matrix. The forecasting procedure is as follows.

First, calculate $\hat{\beta }$ from the initial values of ***θ*** and *σ*^2^ computed from the basis matrix ***H*** and the training data ***D***, using the following equation:

(14) $$\hat{\beta }\left(\theta ,\sigma^{2}\right)={\left[H^{T}{\left[K+\sigma^{2}I\right]}^{-1}H\right]}^{-1}H^{T}{\left[K+\sigma^{2}I\right]}^{-1}F_{x}$$

Then, estimate ***θ*** and *σ*^2^ that maximize the log-likelihood function expressed in the following equation:

(15) $$\;\log p\left(F_{x}\right)=-\frac{1}{2}{\left(F_{x}-H\hat{\beta }\right)}^{T}{\left[K+\sigma^{2}I\right]}^{-1}\left(F_{x}-H\hat{\beta }\right)-\frac{n}{2}\log2\pi -\frac{1}{2}\log\left|K+\sigma^{2}I\right|$$

Using the estimated ***β***, ***θ***, and *σ*^2^, find the probability distribution of the latent variable $F_{x\mathrm{test}}{}^{*}$ in the GPR and calculate the probability distribution of the predicted values using the following formula:

(16) $$\mu =k_{*}{\left[K+\sigma^{2}I\right]}^{-1}F_{x}-H\beta$$

(17) $$\Sigma =k_{**}-k_{*}{\left[K+\sigma^{2}I\right]}^{-1}k_{*}{}^{T}$$

(18) $$p\left(F_{x\mathrm{test}}{}^{*}|f_{\mathrm{test}},D\right)=N\left(\mu ,\Sigma \right)$$

where K, $k_{*}$, and $k_{**}$ are kernel matrices and $k\left(f^{i},f^{j}\right)$ is the kernel function.

(19) $$K=\left[\begin{array}{cccc} k\left(f^{1},f^{1}\right) & k\left(f^{1},f^{2}\right) & \cdots & \;\;\;k(f^{1},f^{n})\\ k\left(f^{2},f^{1}\right) & k\left(f^{2},f^{2}\right) & \cdots & \;\;\;k(f^{2},f^{n})\\ \vdots & \vdots & \ddots & \vdots \\ k\left(f^{n},f^{1}\right) & k\left(f^{n},f^{2}\right) & \cdots & k\left(f^{n},f^{n}\right) \end{array}\right]$$

(20) $$k_{*}=\left[\begin{array}{cccc} k\left(f^{1},f^{1}\right) & k\left(f^{1},f^{2}\right) & \cdots & \;\;\;k(f^{1},f^{n_{\mathrm{test}}})\\ k\left(f^{2},f^{1}\right) & k\left(f^{2},f^{2}\right) & \cdots & \;\;\;k(f^{2},f^{n_{\mathrm{test}}})\\ \vdots & \vdots & \ddots & \vdots \\ k\left(f^{n},f^{1}\right) & k\left(f^{n},f^{2}\right) & \cdots & k\left(f^{n},f^{n_{\mathrm{test}}}\right) \end{array}\right]$$

(21) $$k_{**}=\left[\begin{array}{cccc} k\left(f^{1},f^{1}\right) & k\left(f^{1},f^{2}\right) & \cdots & \;\;\;k(f^{1},f^{n_{\mathrm{test}}})\\ k\left(f^{2},f^{1}\right) & k\left(f^{2},f^{2}\right) & \cdots & \;\;\;k(f^{2},f^{n_{\mathrm{test}}})\\ \vdots & \vdots & \ddots & \vdots \\ k\left(f^{n_{\mathrm{test}}},f^{1}\right) & k\left(f^{n_{\mathrm{test}}},f^{2}\right) & \cdots & k\left(f^{n_{\mathrm{test}}},f^{n_{\mathrm{test}}}\right) \end{array}\right]$$

The squared exponential kernel was expressed as follows:

(22) $$k\left(f^{i},f^{j}\right)=\sigma_{f}{}^{2}\exp\left[-\frac{1}{2}\frac{{\left(f^{i}-f^{j}\right)}^{T}\left(f^{i}-f^{j}\right)}{\sigma_{l}^{2}}\right]$$

where $\sigma_{f}$ and $\sigma_{l}$ are the standard deviations of the training data of ***f*** and the characteristic length scale, respectively. $\sigma_{f}$ and $\sigma_{l}$ need to be greater than 0 and can be enforced by the unconstrained parametrization vector ***θ***= (log$\sigma_{f}$, log$\sigma_{l}$)^T^. MLR and GRP models were trained using the Matlab ver. 9.11 (Mathworks, Natick, MA, USA) Statistics and Machine Learning Toolbox.

Prediction models for each movement and all movements were created by machine learning using all subject data. Therefore, 8 persons × 10 steps = 80 cases were used for each movement model, and 8 persons × 10 steps × 3 movements = 240 cases were used for all movement models. Leave-out-one cross-validation was used. That is, a regression model was created using one step of the measured data as test data ***T*** and the remaining data as training data ***D***. By changing the trials used as test data and training data, the prediction accuracy of all trials used to create the model was determined, and the average of the predictions was calculated.

The mean absolute error (MAE), the %RMSE, and the degree-of-freedom-adjusted coefficient of determination R^2^ were used as evaluation indices for the prediction accuracy of the three-directional GRFs. The %RMSE was obtained by normalizing the RMSE by the range between the maximum and minimum values of the measured GRF values. For example, the MAE, %RMSE, and the R^2^ in the *x* direction are expressed by the following equations:

(23) $$\mathrm{MAE}=\frac{1}{n}\;\overset{n}{\underset{j=1}{\sum }}\left|F{\;}_{x\mathrm{test}}^{j}-\hat{F}{\;}_{x\mathrm{test}}^{j}\right|\;$$

(24) $$\%\mathrm{RMSE}=\sqrt{\frac{\sum_{j=1}^{n}{\left(F{\;}_{x\mathrm{test}}^{j}-\hat{F}{\;}_{x\mathrm{test}}^{j}\right)}^{2}}{n}}\frac{100}{F_{x\max}-F_{x\min}}$$

(25) $$R^{2}=1-\frac{\sum_{j=1}^{n}{\left(F{\;}_{x\mathrm{test}}^{j}-\hat{F}{\;}_{\mathrm{xtest}}^{j}\right)}^{2}}{\sum_{j=1}^{n}{\left(F{\;}_{x\mathrm{test}}^{j}-{\bar{F}}_{x\mathrm{test}}\right)}^{2}}\frac{n-1}{n-k-1}$$

where $F{\;}_{x\mathrm{test}}^{j}$ and $\hat{F}{\;}_{x\mathrm{test}}^{j}$ are the measured GRF used for the test data and the predicted GRF in the *x* direction (1 ≤ *j* ≤ n), respectively, and ${\bar{F}}_{x\mathrm{test}}$ is the mean value of the measured GRF used for the test data. Note that the MAE was calculated using the $F{\;}_{x\mathrm{test}}^{j}$ and $\hat{F}{\;}_{x\mathrm{test}}^{j}$ divided by the participant’s body mass.

## 3. Results

### 3.1. Estimation of GRFs Using the Data of Each Movement

**Figure 6:**
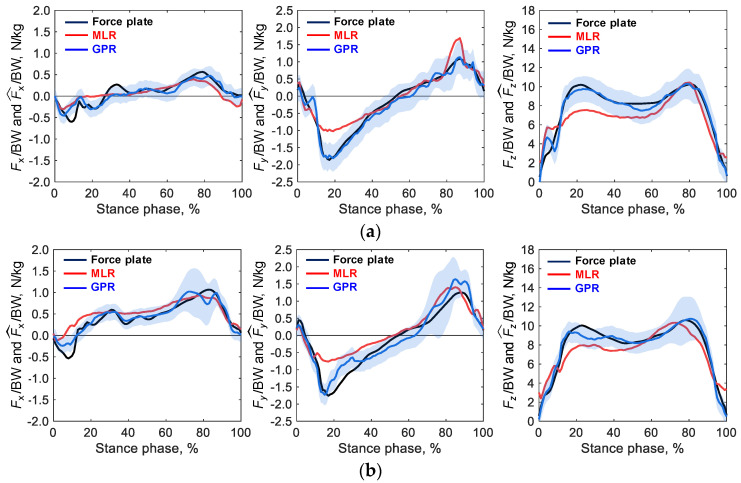

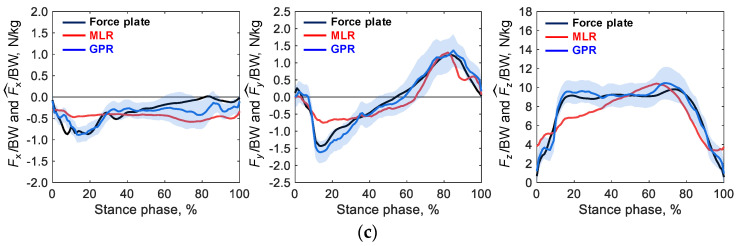
Examples of time-series changes in the predicted and measured ground reaction force (GRF) values in the test trials for each movement, using the regression model trained on the data of each movement for (**a**) straight walking, (**b**) side-step turning, and (**c**) cross-step turning. The horizontal axis indicates the normalized period with 0% for heel ground contact and 100% for toe-off, and the vertical axis indicates the measured GRFs and predicted GRFs divided by the participant’s body mass. The solid black line shows the GRF values measured with the force plate, the solid red line shows the predictions by multiple linear regression (MLR), the solid blue line shows the predictions by Gaussian process regression (GPR), and the light blue shaded area shows the 95% confidence interval of the prediction by GRP.

Figure 6 shows examples of time-series changes in the predicted and measured GRF values in the test trials for each movement, using the regression model trained on the data of each movement. The figure shows that in the *y* and *z* directions, the GPR model accurately predicted GRFs measured with the force plate throughout the stance phase for each movement. Conversely, the prediction using multiple regression analysis tended to be less accurate in the first half of the stance phase. Estimation accuracy was lower in cross-step turning (Figure 6c) than in straight walking (Figure 6a) and side-step turning (Figure 6b).

Table 1 presents the mean ± standard deviation of the prediction error, MAE, %RMSE, and R^2^ for each movement using the regression model trained on the data of all participants. The table shows that for both straight walking and side-step turning, the prediction of the GRF component in any direction had a %RMSE of less than 15%, regardless of the regression model. The GPR model had higher estimation accuracy than the MLR model, with a %RMSE of less than 15% in any direction of each type of gait, and the successful prediction was within the error range acceptable for clinical application. For straight walking and side-step turning, the GPR model showed good estimation accuracy, with a %RMSE of less than 10% and R^2^ of greater than 0.7, regardless of the direction. %RMSE and R^2^ was lower in the *x* direction than in the *y* and *z* directions; however, the MAE was lower in the *x* direction than in the *y* and *z* directions. In the case of cross-step turning, the %RMSE exceeded 15% for the prediction of the GRF component in the *x* direction using the MLR model.

### 3.2. Estimation of GRFs Using Data of All Movements

Table 2 shows the mean ± standard deviation of the MAE, %RMSE, and R^2^ for each regression model trained on the data of all movements. The GPR model achieved lower MAE and %RMSE than the MLR model. Furthermore, the GPR model achieved a % RMSE of less than 10% in the *y* and *z* directions. However, in the *x* direction, the error was greater than 15% for both regression models. Thus, as with the models trained on each movement, the estimation accuracy was higher with the GRP model than with the MLR model. In addition, comparing these data with the data in Table 1, it was noted that the estimation accuracy was higher with the models trained specifically on the data of each movement than with the models trained on the data of all movements.

## 4. Discussion

In the three-directional GRF prediction using our shoe sole sensor system and machine learning, the %RMSE between the predicted and measured values was less than 15% for both regression models in the *y* and *z* directions, and the prediction accuracy of the GRFs was considered sufficient for practical use [6]. The error of the prediction using the model for straight walking only was smaller than that of the conventional three-directional GRF prediction using pressure sensors [20], indicating the practical feasibility of GRF prediction using our shoe sole sensor system. Alternatively, in the *x* direction, the %RMSE exceeded 17% for the prediction using the model of all movements, indicating that the prediction using the model of all movements is not sufficiently accurate.

### 4.1. Difference in Prediction Accuracy by Direction

Our results showed that the %RMSE for GRFs tended to be larger in the *x* direction than in the *y* and *z* directions, regardless of the type of training data, motion, or regression model used. However, the MAE in the *x* direction for each movement model was lower than that in the *y* and *z* directions. The MAE in the *x* direction for all movement model was comparable with that in the *y* direction and lower than that in the *z* direction. This is because the absolute GRF values were larger in the *y* and *z* directions than in the *x* direction, resulting in a smaller %RMSE. In other studies [19,21], the %RMSE was calculated using the participant’s body mass to normalize the RMSE value, and in this case, the %RMSE tended to be smaller in the *x* direction than in the *y* and *z* directions.

In addition, in this study, the directions of side-step turning and cross-step turning were reversed in all motion models, so the data of motion with GRFs in the opposite directions in the *x* direction were mixed. There was only one sensor in the heel, so the *x*-directional GRF output from the heel to the midfoot was not sufficient to distinguish between side-step and cross-step turning. Therefore, when the regression model was used for each movement, the prediction accuracy in the *x* direction for both side-step and cross-step turning improved compared with that in the all movement model, as shown in Table 1 and Table 2. Based on these findings, it is considered effective to use different regression models to distinguish between different walking motions in order to improve the prediction accuracy of *x*-directional GRFs during different walking motions.

### 4.2. Difference in Prediction Accuracy by Movement

As shown in Table 1, the prediction accuracy in the *x* direction tended to be lower for cross-step turning than for straight walking and side-step turning, regardless of the regression model. Cross-step turning is a difficult movement in which the body’s center of mass tends to deviate from the base of support area and the base of support is narrow, making it necessary to change direction while maintaining body balance during the movement [34,35]. We determined the between-participant and within-participant standard deviations of the GRFs divided by the participant’s body mass throughout the stance phase in the *x* direction. The mean value of the between-participant standard deviation of the GRF during cross-step turning (0.221) was larger than that during straight walking (0.148) and side-step turning (0.151). The mean value of the within-participant standard deviation of the GRF during cross-step turning (0.183) was larger than that during straight walking (0.089) and side-step turning (0.121). Therefore, the variation of GRFs in the left-right direction during cross-step turning within-participant and between participants was larger than that during straight walking and side-step turning. The prediction accuracy for side-step turning was equivalent to that for straight walking, as shown in Table 1. This is thought to be due to the fact that the side-step turning has a larger base of support in the left-right direction [34,35], making it easier to maintain body balance in the left-right direction.

### 4.3. Difference in Prediction Accuracy by Regression Models

As shown in Table 1 and Table 2, the error was smaller in the GPR model than in the MLR model, as expected. GPR, which is a nonlinear regression, allows the construction of complex models dealing with nonlinear relationships between objective variables and explanatory variables that cannot be handled by multiple regression analysis, which is a linear regression. In this study, we did not use methods, such as neural networks [36], because of the small data set, but such machine learning methods are effective if the training data set is made larger by increasing the number of participants and the number of steps. However, the results of this study indicate that GPR can perform accurate learning even with relatively small data sets.

### 4.4. Study Limitations

Some limitations of the current study should be considered. First, the number of participants was small, and the representation of genders was uneven. Thus, our results may not be generalizable to a broader population. In addition, studies with a large sample size will provide improved GRF prediction accuracy. Second, there were only three types of walking movements. It is considered necessary to create prediction models with a data set that considers various movements performed in daily life in addition to straight walking, side-step turning, and cross-step turning.

## 5. Conclusions

The regression model trained for each motion, except for MLR in cross-step turning, showed less than 15% prediction error (%RMSE) regardless of the direction. The three-directional GRFs during walking predicted by the shoe sole sensor system showed relatively good agreement with the GRFs measured with the force plate, indicating the practical applicability of the shoe sole sensor system for gait analysis. However, the prediction error in the *x* direction (%RMSE) for the whole motion model exceeded 15%. Compared with straight walking and side-step turning, cross-step turning tended to have a lower prediction accuracy in the *x* direction, regardless of the regression model.

The prediction of three-directional GRFs using our shoe sole sensor system and machine learning could be used in a wide range of practical applications, such as in the sports field and for gait analysis at rehabilitation facilities and in daily life, taking advantage of its portability and accuracy in prediction.

## Figures and Tables

**Table 1:** Results of MAE, %RMSE, and R^2^ for each regression model trained on the data of each movement.

Type of Gait	Regression Model	*x*			*y*			*z*		
		MAE	%RMSE	R^2^	MAE	%RMSE	R^2^	MAE	%RMSE	R^2^
Straight walking	MLR	0.067 ± 0.044	12.8 ± 4.3	0.551 ± 0.217	0.121 ± 0.104	9.5 ± 3.3	0.819 ± 0.071	0.617 ± 0.432	10.8 ± 4.5	0.682 ± 0.161
	GPR	0.047 ± 0.034	9.8 ± 2.7	0.706 ± 0.215	0.111 ± 0.090	6.2 ± 1.4	0.916 ± 0.066	0.237 ± 0.209	4.9 ± 2.2	0.917 ± 0.067
Side-step turning	MLR	0.102 ± 0.063	12.1 ± 2.6	0.673 ± 0.199	0.196 ± 0.160	11.3 ± 3.1	0.743 ± 0.193	0.460 ± 0.345	11.1 ± 4.4	0.674 ± 0.165
	GPR	0.072 ± 0.059	9.7 ± 2.2	0.755 ± 0.251	0.129 ± 0.187	7.5 ± 1.7	0.840 ± 0.458	0.195 ± 0.171	4.9 ± 1.7	0.922 ± 0.067
Cross-step turning	MLR	0.106 ± 0.092	20.1 ± 5.5	0.281 ± 1.629	0.178 ± 0.129	11.1 ± 2.9	0.752 ± 0.124	0.517 ± 0.340	12.3 ± 4.9	0.606 ± 0.176
	GPR	0.062 ± 0.055	13.8 ± 2.2	0.378 ± 0.541	0.124 ± 0.114	7.6 ± 1.2	0.867 ± 0.156	0.177 ± 0.186	5.1 ± 2.0	0.911 ± 0.107

Abbreviations: MAE, mean absolute error; %RMSE, percentage root-mean-square error; MLR, multiple linear regression; GPR, Gaussian process regression.

**Table 2:** Results of MAE, %RMSE and R^2^ for each regression model trained on the data of all movements.

Regression Model	*x*			*y*			*z*		
	MAE	%RMSE	R^2^	MAE	%RMSE	R^2^	MAE	%RMSE	R^2^
MLR	0.222 ± 0.221	24.4 ± 6.6	−0.943 ± 2.478	0.181 ± 0.143	11.3 ± 3.1	0.749 ± 0.178	0.523 ±0.383	11.8 ± 4.7	0.630 ± 0.176
GPR	0.120 ± 0.115	17.5 ± 3.5	−0.054 ± 1.501	0.115 ±0.102	6.9 ± 1.0	0.894 ± 0.122	0.198 ± 0.168	5.2 ± 2.2	0.913 ± 0.063

Abbreviations: MAE, mean absolute error; %RMSE, percentage root-mean-square error; MLR, multiple linear regression; GPR, Gaussian process regression.

## Data Availability

The data including graphs and table within this paper are available from the corresponding author upon reasonable request.
